# An inertial‐based spiral device for separating rare tumor cells from liquid biopsies

**DOI:** 10.1002/btm2.70175

**Published:** 2026-09-23

**Authors:** Isidora Panez‐Toro, Laurent Griscom, Dominique Heymann, Javier Muñoz‐García

**Affiliations:** ^1^ Biological Sciences and Biotechnologies Unit Nantes Université, CNRS, UMR6286, US2B Nantes France; ^2^ Institut de Cancérologie de l'Ouest Tumor Heterogeneity and Precision Medicine Laboratory Saint‐Herblain France; ^3^ CNRS, INSERM, Biosit, UAR 3480 US_S 018, BIM3D Core Facility University of Rennes Rennes France; ^4^ School of Medicine and Population Health University of Sheffield Sheffield UK

**Keywords:** circulating tumor cells, inertial microfluidics, liquid biopsy, microchip, osteosarcoma, particles and cell separation

## Abstract

Early detection of primary tumors and metastatic processes increases the success of anti‐tumor therapies and favors good prognoses. Within the current range of methods available to detect the presence of tumors, liquid biopsy is receiving particular attention as it is a minimally invasive and quick procedure. Identification of cancer elements such as circulating tumor cells (CTCs) constitutes valuable information about tumor biology. However, this is a challenging process due to the rarity of those cells in corporal fluids such as blood (CTCs <10 cell/10 mL blood). Microfluidics, an affordable, sensitive, user‐friendly and rapidly evolving technology, has significantly provided advances in the detection and isolation of rare cancer cells. One of the main strategies currently employed is the development of inertial focusing devices with spiral geometries. In these systems, hydrodynamic forces are used to focus particles as a function of their size without additional mechanical or electronic equipment. Specifically, the interactions between inertial lift forces and Dean vortices determine the equilibrium position of particles as a function of their size, enhancing their sorting. Here, by combination of viscoelastic and inertial microfluidics approaches, we have developed a spiral device for isolation of CTCs from liquid biopsies. Our device was able to isolate and enrich with high efficiency osteosarcoma cells presented in a low number from whole blood samples. This device constitutes a promising tool for isolation, characterization and potential culture of tumor cells from patient samples and improve personalized medicine.

## Graphical abstract

Development of an elliptic inertial‐based microdevice as a promising tool for isolation, characterization, and potential culture of CTCs from liquid biopsies. This new microsystem may help with deciphering tumor properties and biomarkers for early cancer detection and development of personalized medicine therapies.

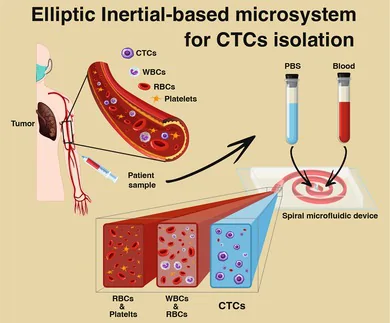

**Translational Impact Statement**

Early detection of cancers substantially improves cancer therapies and patients' responses. In this sense, the use of liquid biopsy techniques constitutes a potential and minimally invasive approach. Here we developed an elliptic microfluidic device for effective detection and isolation of circulating tumor cells (CTCs) from blood biopsies with minimal sample perturbation and a fast procedure (less than 30 min). This device can be used as a standard tool for real‐time monitoring of patient tumor evolution and effectiveness of anti‐cancer therapies in clinics.

## 1 INTRODUCTION

According to last worldwide estimations, by 2050 more than 30 million people will be affected by cancer. Comparing to 2022, a global increase of 75% of cancer cases and 90% of death by this disease are projected, mostly coming from low‐Human Development Index countries.^1^ This quick increase in global cancer burden reflects an aging population and exposure to risk factors associated with socio‐economic development. Based on these predictions, the urgency of improvement in cancer early detection and diagnosis is evident. Despite the increase of potential anti‐cancer therapies, major advances in cancer survival rate are attributed to prevention programs and early cancer detection methods.^2^ In this direction, early detection of cancers is undergoing a continuous revolution during the last decades due to the development of multi‐omic approaches that integrate genomic, transcriptomic, proteomic, metabolic, advanced microscopy, liquid biopsy and, lately, artificial intelligence analytics technologies, that are substantially improving cancer therapies and patients' responses.^3^

Liquid biopsy^4^ techniques, including detection of circulating cancer elements such as DNA, ARN, extracellular vesicles (EVs), or cells, constitute a minimally invasive methodology permitting real‐time monitoring of anti‐cancer therapies and tumor evolution.^5^ Within the panel of approaches developed for liquid biopsy analysis,^6^ microfluidics technology emerges as an appealing tool.^7^ Its flexibility in architecture design, the affordable cost for microfluidic chips production and the use of minimal sample or reagent volume makes this technology a promising platform for detection, isolation or enrichment of rare biological samples presents in liquid biopsies. Thus, its capability to integrate optical, electric, chemical, or mechanical sensors into miniaturized devices makes it a powerful analytical tool.^8^

Within the scope of rare cancer elements, circulating tumor cells (CTCs) constitute a major target in the development of microfluidics devices.^9^ CTCs are the basis of the tumor dissemination and metastatic development processes.^10^ Importantly, CTCs are characterized by a heterogeneous population of tumor cells with high mechanical and metabolic plasticity, which favor cell dissemination, invasion, and colonization of target tissues.^11^ Once in the blood stream or lymph system, CTCs are directly exposed to a hostile environment including immune and blood cell interactions including high shear stresses, which all compromise tumor cell survival.^12^ It has been estimated that the presence of live CTCs in blood is less than 1 h.^13^ Additionally, the number of CTCs present in the blood of patients affected by cancer is considerably low (<10 cell/10 mL blood) compared to blood cells,^14^ making the detection of CTCs a challenging task. However, it is generally accepted that CTCs present a cell size that is slightly larger than white blood cells, with lower deformability.^15^ This feature has been used to discriminate CTCs from the rest of the cells present in blood in other studies.^16^

Microfluidics emerged as an affordable and powerful technology which significantly provided advances in the detection and isolation of particles.^17^ In 1961, Segré and Silberberg described inertial focusing as the phenomenon by which randomly distributed particles migrate to ordered lateral equilibrium positions under laminar flow in straight channels.^18^ Since then, inertial focusing has been used in a variety of microfluidics techniques to separate particles or cells as a function of their size. A detailed theoretical description of inertial focusing can be found in.^19^ Forty years later, in 1999, Asmolov demonstrated that particles confined in a parabolic flow are subjected to lift forces, the shear‐induced lift force that pushes particles away from the channel centerline and the wall‐induced lift force that repels the particles away from the wall, which determined the focusing of particles at the equilibrium position.^20^ In 2007, Di Carlo theoretically and practically demonstrated the importance of microchannel geometry (the hydraulic diameter) and the Dean number parameters paramount to focusing and ordering particles in a single beam.^21^ Finally, in 2009 Gossett and Di Carlo showed that the addition of curvature to microchannels generated secondary drag forces that improved particle focusing and ordering.^22^ Based on these theoretical and practical advances, several groups have developed spiral microfluidics devices for particle separation and cell separation. The spiral geometry generates a secondary flow that imparts a new set of drag forces which impose lateral equilibrium position within the microchannel of particles as a function of their size.^23^ Thus, spiral microdevices are currently used for cell filtration, including yeast or mammalian cells^24^, ^25^ and cell isolation, from bacteria to microalgae and to tumor cells.^26^, ^27^, ^28^, ^29^, ^30^, ^31^

In the present work, we have developed two spiral microfluidic devices, one with Archimedean (circular) shape and another one with an elliptical shape for isolation and enrichment of tumor cells present in blood samples. Each microfluidic device presents a particular spiral shape, round or oval, but shares consistent architectural design features (main‐channel cross‐sections, inlets and outlets). First, particles of different sizes have been used to analyze the influence of flow rate and fluid density in size particle isolation, from single to mixed particle solutions. Then, spiral devices were challenged for tumor cell isolation in controlled solutions and complex solutions like blood. Finally, the elliptical spiral design was tested and compared to the circular design and shows improved isolation and enrichment performance without the need for classical pre‐treatment and manipulation of blood.

## 2 RESULTS

### 2.1 Effect of symmetrical or asymmetrical flow rates on individual particle distribution in spiral microdevices

**FIGURE 1:**
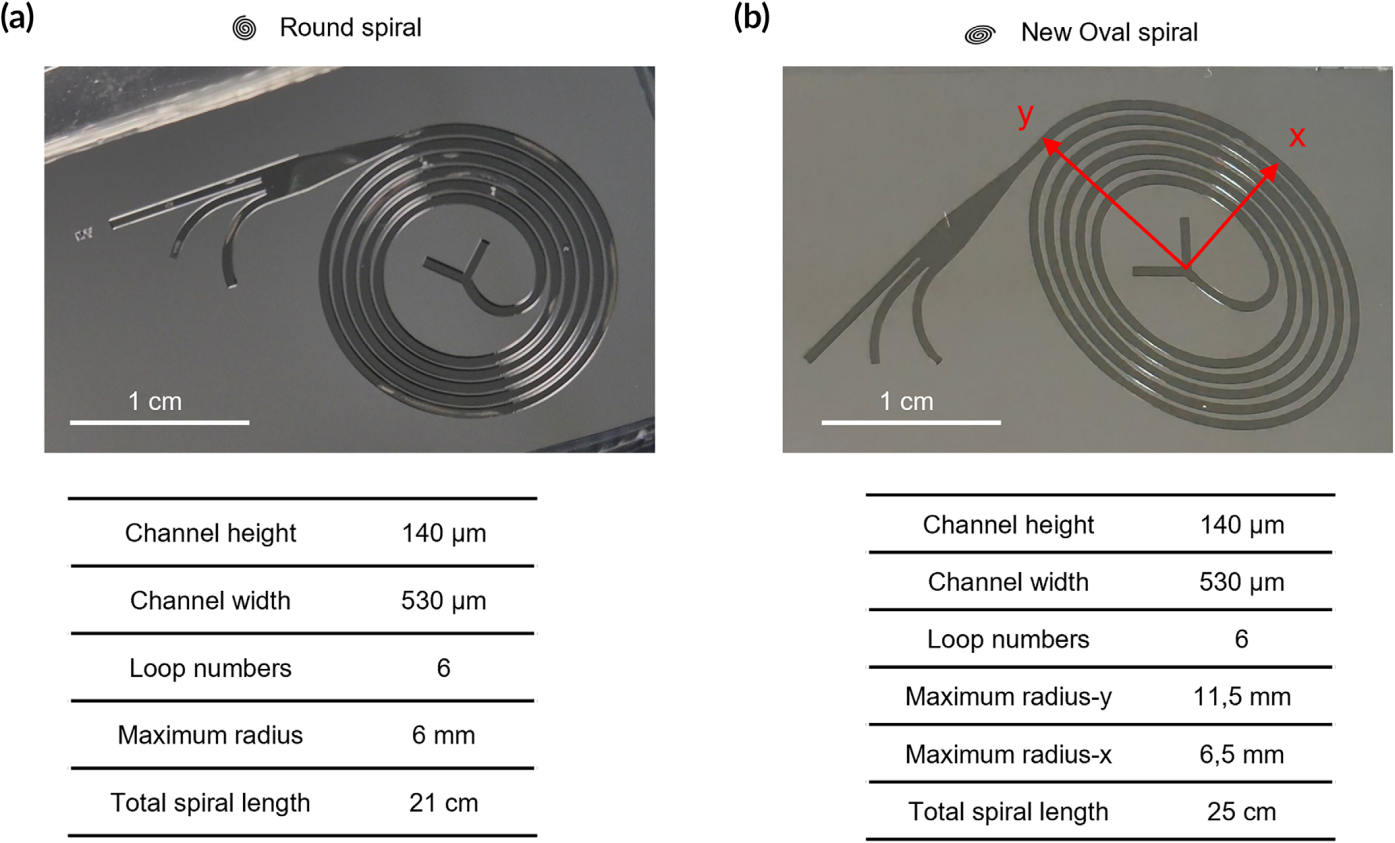
Inertial round and oval spiral microfluidics devices. (a) Round spiral microsystem consisting of six loops spiral in the counterclockwise direction with two inlets at the center of the microchannel and three outlets outside in a perpendicular position with respect to the inlets. The main channel has a total length of 21 cm with a rectangular cross‐section, measuring 530 μm in width and 140 μm in height, resulting in a low aspect ratio (H/W = 0.26). The radius of the round spiral is 6 mm with a loop spacing of 220 μm. (b) Oval spiral microsystem has a similar configuration to the round spiral with six loop spirals in a counterclockwise direction and identical inlets and outlet exits. The main channel has a total length of 25 cm with a rectangular cross‐section measuring 530 μm in width and 140 μm in height. The short radius in the *x*‐axis has a size of 6.5 mm and the long radius in the y‐axis has a size of 11 mm. The loop spacing at the short *x*‐axis of the oval is 220 μm, whereas the long axis is 500 μm. Scale bar, 1 cm.

We generated two different spiral microfluidic devices based on inertial forces for tumor cells separation and enrichment from blood samples (Figure 1). Both microdevices present identical inlets, main spiral channel cross‐section, and exits (see Section 4). The major difference between them is the shape of the spiral: one Archimedean (named round spiral) and the other with an elliptical shape (named oval spiral). Under this configuration, and compared to the round geometry, the oval shape introduces a radius of curvature oscillation within each loop, which enhances the inertial and secondary forces that could help in the redistribution of cells and particles and their isolation.

To mimic the cellular components presented in a blood sample, three polystyrene microsphere suspensions with different sizes (10, 13 and 26 μm) were used. Note that tumor cell sizes are heterogeneous, and the use of polystyrene particles represents a simplified model of this complexity. The smaller particles (lower than 10 μm) represented the behavior and distribution of smaller elements in blood (platelet and red blood cells (RBCs)),^32^, ^33^ medium particles (13 μm) symbolized the average size of white blood cells (WBCs),^34^ whereas big particles (26 μm) can be associated with larger WBCs and tumor cells.^16^

To simulate the fluidic properties of blood, particles were resuspended in a solution of BSA at 30% in PBS. This solution presents similar values of density (1094 kg/m^3^) and viscosity (0.005 Pa⋅s) to those exhibited in blood at physiological conditions (1060 kg/m^3^ and 0.0035 Pa⋅s, respectively,^35^).^36^, ^37^, ^38^

**FIGURE 2:**
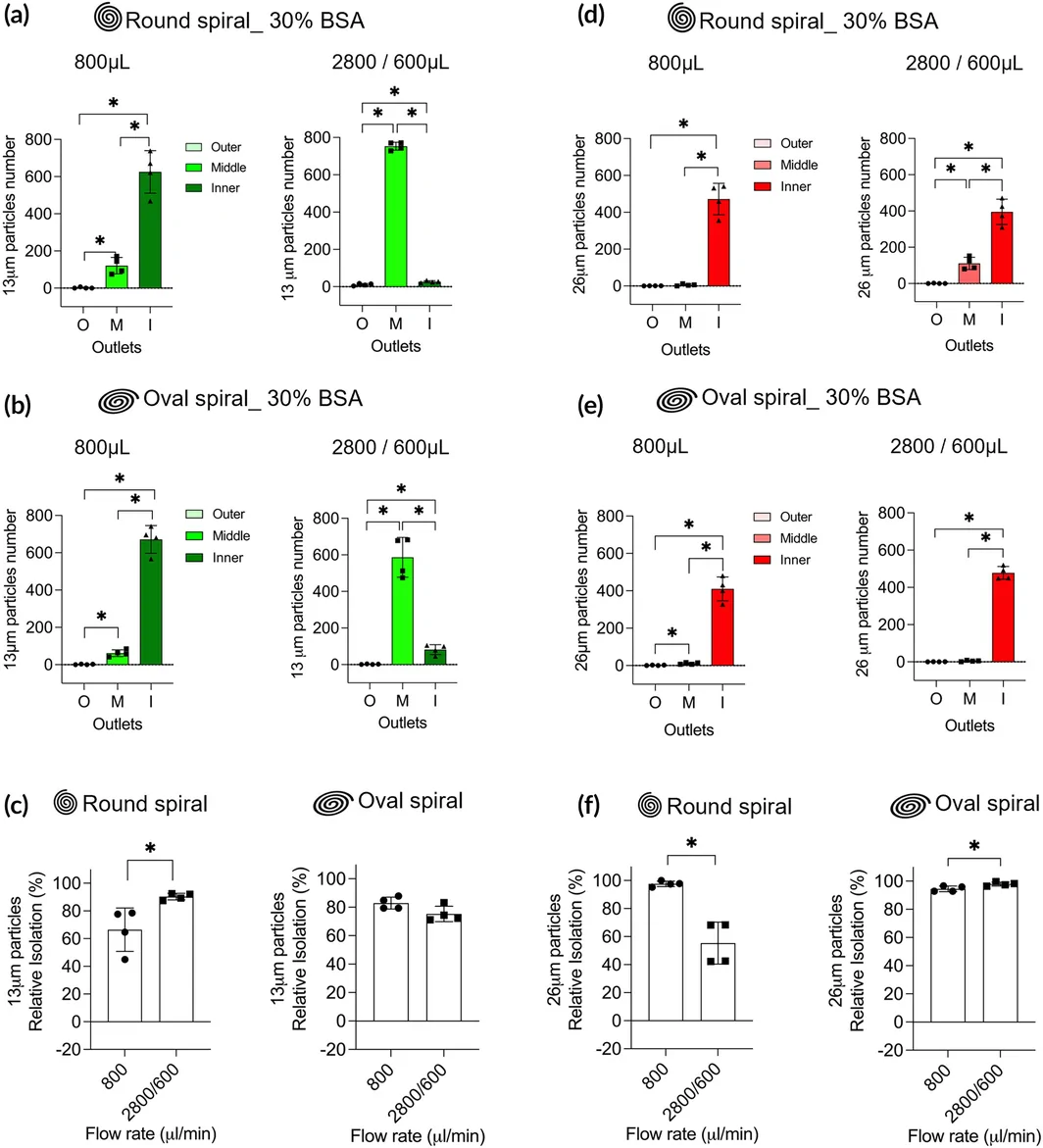
Individual particle isolation at different flow rates in round and oval spirals. (a) Distribution of 13 μm microspheres subjected to symmetrical or asymmetrical flow rates in the round spiral microdevice. 2.5 mL of a solution containing 800 particles of 13 μm size in BSA 30% was injected simultaneously with 2.5 mL of PBS into the round spiral at an equal flow rate of 800 μL/min (symmetric flow, left panel). Particles were collected from different outlets; images were obtained and particles counted with a fluorescent microscope. Under symmetric flow rate, the 13 μm particles were significantly located within the inner outlet, whereas under asymmetric flow rate, particles were collected at the middle outlet (asymmetric flow, right panel). (b) Distribution of 13 μm microspheres subjected to symmetrical or asymmetrical flow rates in the oval spiral microdevice. Similar analysis to (a) was performed, showing similar distribution behavior to the round spiral. (c) Quantification of relative isolation of 13 μm particles in the main collector channel compared to the other two outlets. (d) Distribution of 26 μm microspheres subjected to symmetrical or asymmetrical flow rates in the round spiral microdevice. Data was analyzed as (a). Twenty‐six micrometers particles were significantly located in the inner outlet for both injection rates. (e) Distribution of 26 μm microspheres subjected to symmetrical or asymmetrical flow rates in the oval spiral microdevice. Data was analyzed as (a). As was shown in (d), particles were significantly located within the inner outlet regardless of the flow rate applied. (f) Quantification of relative isolation of 26 μm particles in the main collector channel compared to the other two outlets. For each test, data are expressed as mean ± standard deviation of four independent experiments. All data were analyzed by unpaired *t*‐test or their nonparametric equivalent Mann–Whitney *U* test. *p‐value <0.05. O: outer outlet. M: middle outlet. I: inner outlet.

First, we evaluated the impact of the flow rate on the distribution and isolation of monodisperse polystyrene microspheres. We tested 13 μm (green color) and 26 μm (red color) individually in the respective microfluidic devices (Figure 2). We based the flow conditions on previous studies on spiral devices,^30^ where two flow regimes were used: a symmetrical flow (two syringes at 800 μL/min each) and an asymmetrical flow rate (one syringe at 2800 μL/min of PBS at the inner input and the other one at 600 μL/min with particle solution at the outer input).

In the case of the round spiral, symmetrical flow rate resulted in a distribution of 13 μm particles concentrated mainly at the inner outlet (Figure 2a, left panel), whereas asymmetrical flow rate induced focusing of 13 μm particles toward the middle outlet (Figure 2a, right panel). Similar behavior was observed in the oval spiral microdevice, with a significant distribution of 13 μm particles focused into the inner outlet when submitted to symmetric flow rate (Figure 2b, left panel) and into the middle outlet when an asymmetric flow rate was applied (Figure 2b, right panel). The percentage of particles in the main collector outlet (middle) compared to the other two outlets was used as an index to reflect the relative particle isolation efficiency. Only significant differences of relative particle isolation between flow rates were found when the round spiral microdevice was used. In this case, asymmetrical injections generated a relative isolation of 90.3% ± 2.4% of particles at the middle outlet whereas for symmetrical injection, only a relative isolation of 66.4% ± 15.65% of particles was obtained in the inner channel (Figure 2c, left panel). No significant differences were found for the oval spiral when symmetric or asymmetric injections were applied, 82.9% ± 4.18% and 75.27% ± 5.45%, respectively (Figure 2c, right panel).

For individual solutions of 26 μm particles no major differences were observed, neither by the flow rate nor the spiral microdevices. Symmetrical flow rates resulted in the distribution of 26 μm particles into the inner exits of both round and oval spiral (Figure 2d,e, left panels). Similarly, asymmetrical flow rates showed a significant distribution of 26 μm particles into the inner exits (Figure 2d,e, right panels). In terms of relative isolation of 26 μm particles, the main collector outlets were always the inner ones. However, in the round spiral a significant better relative isolation was obtained when symmetric flow rates were applied (97.63% ± 1.96%) than the asymmetric injection (55.33% ± 14.97%) (Figure 2f, left panel). In the case of the oval device, the relative isolation of particles at the inner outlets was higher than 90%, with significant better relative isolation when asymmetric flow was used (97.91% ± 1.36%) with respect to symmetrical injection (94.50% ± 1.98%) (Figure 2f).

Taken together, our data from individual particle solutions suggested that an asymmetric flow rate could be the better injection method for differentially focusing 13 μm particles (middle outlet) apart from the 26 μm particles (inner outlet), with better particle isolation when the oval spiral was used. However, for both microsystems, the use of a symmetrical flow rate did not discriminate individual particle solutions distribution into different outlets.

### 2.2 Fluid density effect on individual particle distribution in spiral microdevices

**FIGURE 3:**
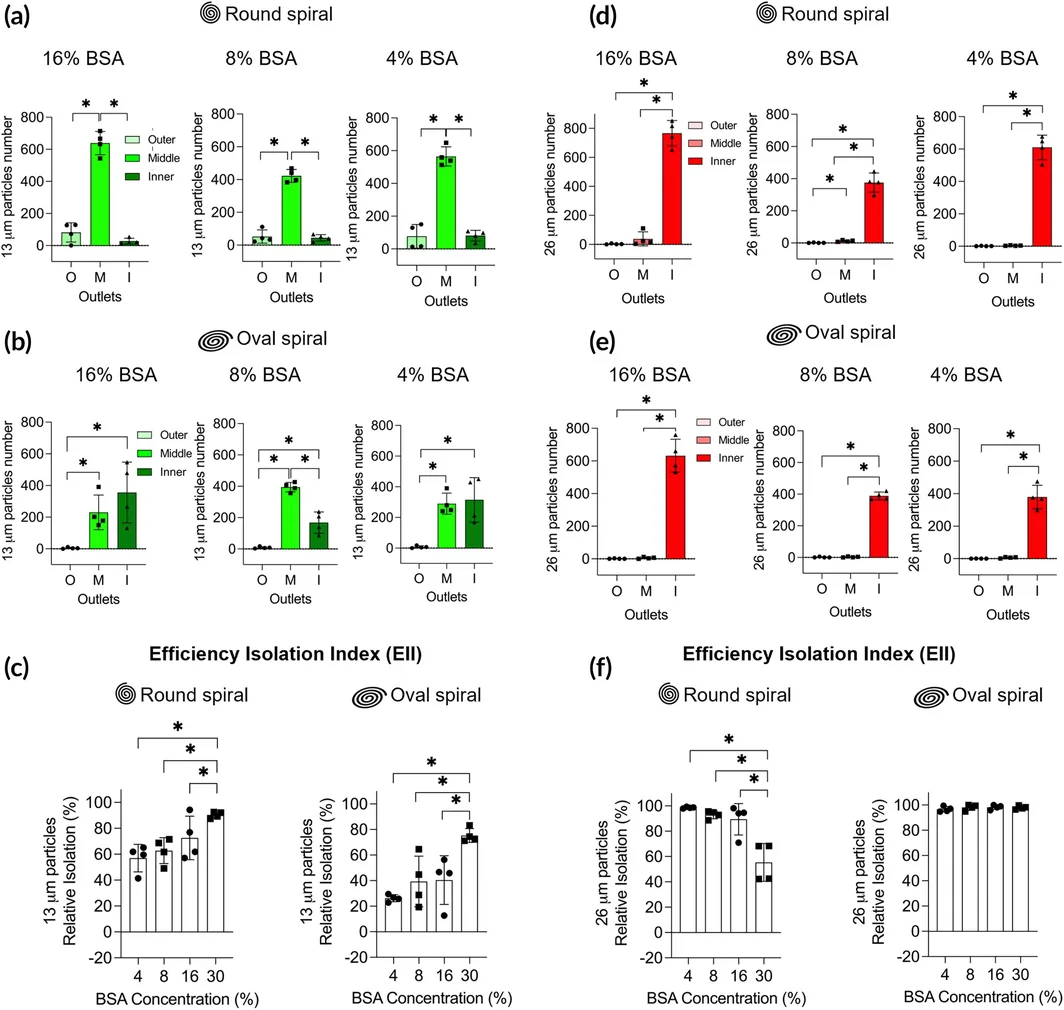
Individual particle distribution in asymmetrical flow rate at different fluid densities in round and oval spirals. (a) Distribution of 13 μm microspheres subjected to BSA densities in the round spiral microdevice. Around 800 particles of 13 μm size (green) were resuspended in different BSA concentrations (16%, 8% and 4%) and injected by asymmetrical flow into the round spiral microdevice. The number of particles collected in each outlet was counted using a fluorescence microscope. Regardless of the density of the BSA particle container, these particles were significantly located at the middle outlet. (b) Distribution of 13 μm microspheres subjected to the same asymmetrical flow rate in the oval spiral microdevice. Analyzed as (a), particles showed a general distribution between the middle and inner outlets. (c) Quantification of relative isolation of 13 μm particles in round and oval devices at different BSA concentrations compared to the outlet at BSA 30%. (d) Distribution of 26 μm microspheres (red) subjected to asymmetrical flow rates in the round spiral microdevice at different BSA concentrations. Identically than for 13 μm beads, the number of 26 μm particles collected in each outlet was counted using a fluorescence microscope. For the three different concentrations of BSA, particles were significantly located at the inner outlet. (e) Distribution of 26 μm microspheres subjected to asymmetrical flow rates in the oval spiral microdevice at different BSA concentrations. As (d), 26 μm microspheres were significantly located at the inner outlet. (f) Quantification of relative isolation of 26 μm particles in round and oval devices at different BSA concentrations compared to the outlet at BSA 30% when summited to asymmetrical flow rate. For each test, data are expressed as mean ± standard deviation of four independent experiments. All particle distributions were analyzed using the Mann–Whitney *U* test and the efficiency isolation index with ordinary one‐way ANOVA and post hoc Holm‐Šídák's multiple comparisons test. *p‐value <0.05. O: outer outlet. M: middle outlet. I: inner outlet.

Studies based on spiral for particles and cells separation in blood tend to dilute blood samples in PBS to facilitate their injection in microfluidics systems.^39^ To evaluate the dilution effect of blood on cell/particle separation in our devices, decreasing concentrations of BSA (16%, 8%, and 4%) were used and compared with the results obtained in the previous section, BSA 30% in asymmetrical flow rate (Figure 3).

In the round spiral device, and as observed for BSA 30%, 13 μm particles were significantly isolated into the middle outlet regardless the percentage of BSA used, with a minor recovery at the inner and outer outlets (Figure 3a). However, the relative particle isolation at BSA 16, 8 and 4% was lower (72.54% ± 16.79%, 62.73% ± 10.02% and 56.96% ± 10.66%, respectively) than the one obtained in the same outlet with BSA at 30% (90.30% ± 2.4%) (Figure 3c, left panel). When oval spiral was challenged at different BSA concentrations, 13 μm particles were collected almost equally at the middle and inner outlets (Figure 3b). This particle behavior was different to the one observed at BSA 30% where particles were mainly focused into the middle outlet (Figure 2b, right panel). Regarding the relative particle isolation index for the same collector outlet (middle), BSA 30% resulted in an index of 75.27 ± 5.45%, higher than those observed at BSA 16% (40.38% ± 19.05%), BSA 8% (39.35% ± 19.83%) or BSA 4% (26.21% ± 2.76%) (Figure 3c, right panel). Those data indicated that reduction of media density did not favor 13 μm particles isolation neither using the round spiral nor the oval spiral. Moreover, for the oval device, particle distribution was split between the middle and inner outlet.

Regarding 26 μm particles isolation at different BSA concentrations, a clear distribution of beads into the inner outlet was obtained whatever the BSA concentration or device was used (Figure 3d,e). In the case of round spiral, the relative isolation index at the main collector outlet (inner) for solution of BSA at 16%, 8% and 4% (89.43% ± 12.43%, 92.98% ± 3.14% and, 98.63% ± 059%, respectively) were significative higher than the one obtained for BSA 30% (55.33% ± 14.97%) (Figure 3f, left panel). However, for the oval spiral the relative isolation index was similar for the different concentrations of BSA tested without significant differences (96.67% ± 2.11% for BSA 4%, 98.09% ± 2.29% for BSA 8%, 98.25% ± 1.76% for BSA16% and, 97.91% ± 1.36% for BSA 30%) (Figure 3f, right panel). These results confirmed that big particle in low density fluids presented higher relative isolation at the inner channel of round spiral microdevices as already demonstrated by Di Carlo,^40^ but in an oval spiral with similar configuration, fluid density does not affect distribution of individual particles of size over 26 μm.

### 2.3 Influence of fluid density in the distribution of a mix of particles of different size

Blood is constituted by a mix of cells/elements of different sizes.^41^ To mimic blood cell behavior in our spiral devices, a mix of particles of different sizes (10, 13, and 26 μm) was used. The trajectory of each particle represents the limits of distribution as a function of their size and can be correlated to different blood cell populations. Smaller particles of 10 μm represent the maximum size and potential distribution of platelets and RBCs. 13 μm particles represent the average size of WBCs. Higher WBCs and tumor cells' trajectories will follow the gap between 13 and 26 μm particles. The particle mixture solution was prepared to mimic the size‐dependent numerical ratio, with 500 particles of 26 μm, 1000 of 13 μm, and 2000 of 10 μm diameter to achieve a 1:2 ratio between consecutive particle sizes to represent analogous concentrations to those found in blood. The increased number of smaller particles reflects their relatively higher abundance compared to the larger ones to perform an accurate model for particle interactions and flow behavior in biological fluids.

**FIGURE 4:**
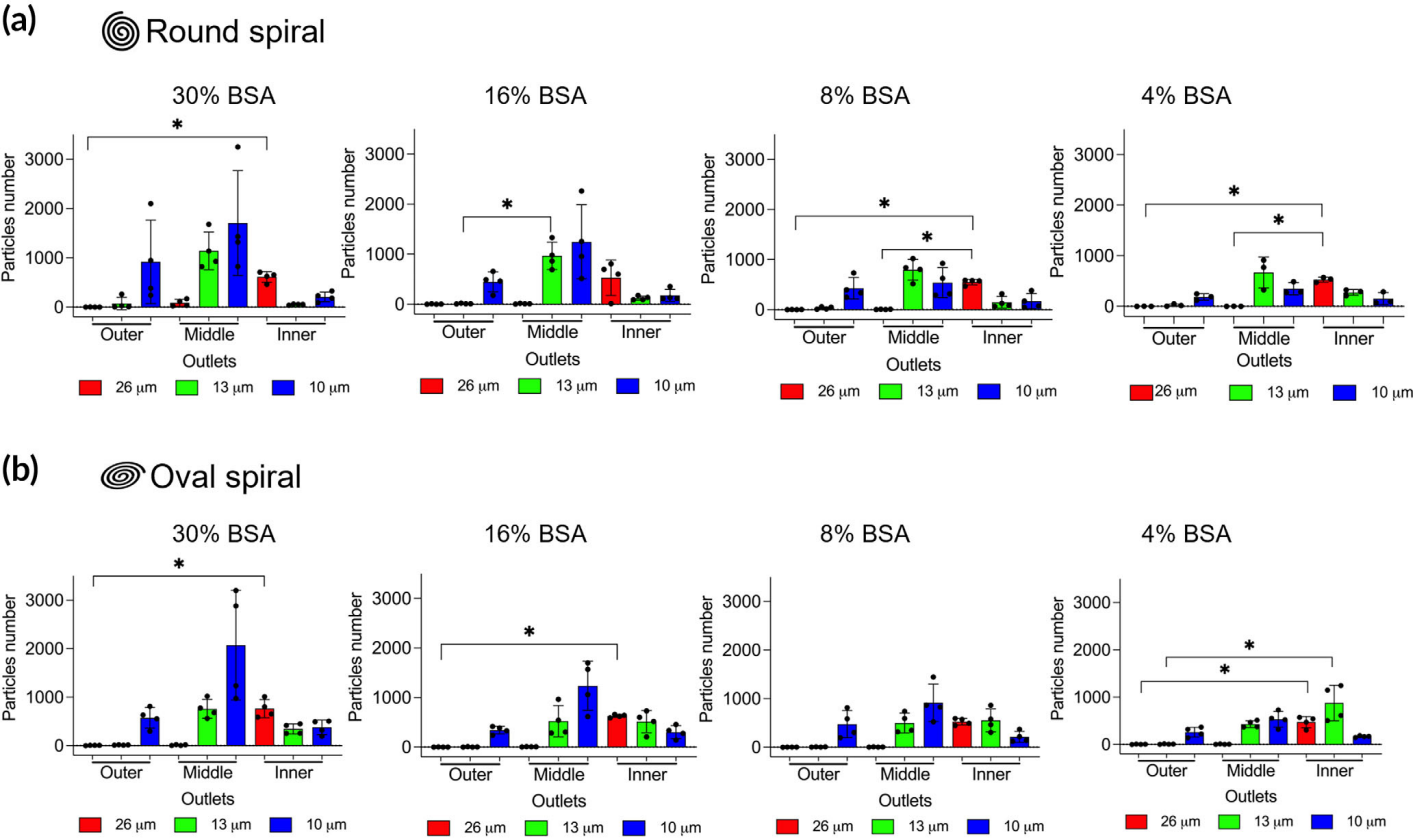
Distribution of mixed‐size particles at different fluid densities in round and oval spirals. (a) Distribution of mix solution containing 500 particles of 26 μm (red), 1000 of 13 μm (green), and 2000 of 10 μm (blue) at different BSA concentrations into the round spiral microdevice at asymmetric flow rate. Particles collected at each outlet were determined by using a fluorescent microscope and specific macro for particles counting in function of size and fluorescence. (b) Distribution of mix solution containing 500 particles of 26 μm, 1000 of 13 μm, and 2000 of 10 μm at different BSA concentrations into the oval spiral microdevice at asymmetric flow rate. Particles were analyzed as (a). For each test, data are expressed as the mean ± standard deviation of four independent experiments. The mixed‐size particles data were treated as paired and analyzed using the Friedman test and post hoc Dunn's multiple comparisons test. *p‐value <0.05.

First, a particle mix solution in BSA 30% was challenged in both spiral microdevices by using asymmetric flow rate. For the round spiral, as showed in Figure 4a (left panel), 10 μm particles were found mainly in the outer and middle channel (blue bars). The relative isolation index at the main isolation outlet (middle) was 33.83% ± 16.04% (Figure S2a, left panel). As previously observed in each individual particle tests section, 13 μm particles were found mainly in the middle outlet (green bar) whereas 26 μm particles were located at the inner one (red bar) (Figure 4a, left panel). The relative isolation index for these particles was 82.77% ± 10.66% and 74.70% ± 21.95% respectively (Figure S2a, middle panel and right panel). In the case of 13 μm particles, the relative isolation index was comparable to the one obtained when analyzed individually (90.30 %± 2.4%, Figure 3e, left panels) however, a significant better relative isolation index was obtained for 26 μm particles from the mix solution than single one (74.70% ± 21.95% vs. 55.33% ± 14.97% respectively). Passing the mix solution through the oval spiral resulted in similar 10 and 26 μm particles distribution (Figure 4b, blue and red bars). For those particles, the relative isolation index using the oval spiral was 32.52% ± 26.66% and 96.14% ± 3.23%, respectively (Figure S2b, left and right panels). However, 13 μm particles were located between the middle and the inner outlets (Figure 4b, green bars), showing a reduction of the expected relative isolation index at the middle outlet of 35.12% ± 22.6% (Figure S2b, mid panel). This could be due to the high occupancy of 10 μm particles at the middle part of the microchannel that could force 13 μm particles to the inner channel.

The reduction of BSA percentage in the mix solution did not favor particle focusing nor better isolation in any of the spiral devices (Figure 4). In the round spiral microdevice, similar particle distribution behavior was observed in BSA 30%, 16%, 8% and 4% (Figure 4a). The final collector outlets for each particle size did not change from those observed when BSA 30% was tested. Regarding the relative isolation index, only a significant difference was observed for 10 μm particles with a reduction of the index at the lower BSA concentrations (9% ± 18.05% for BSA 4% and 0.38% ± 3.67% for BSA 8% vs. 30.21% ± 11.51% for BSA 16% and 33.83% ± 16.04% for BSA 30%) (Figure S2a, left panel). For the oval spiral microdevice, reduction of BSA to 16% or 8% did not alter general particle distribution compared to the mix solution at BSA 30% (Figure 4a). Moreover, as observed for the individual particle assay (Figure 3b, right panel), reduction of BSA to 4% in the oval spiral induced a displacement of 13 μm particles to the inner channel (Figure 4b, last panel). Regarding the relative isolation index obtained in the oval spiral device, no significant differences were observed for each particle (Figure S2b).

These results indicate that, in our spiral devices configuration, particles smaller than 10 μm tend to spread in all the outlets, generating a residual contamination in the outlets where particles of interest are located. In fact, these observations are consistent with the streamline in which the particle‐size focuses respected the relationship between particle size and channel size exposed by Di Carlo et al.^21^ Considering this relationship and the dimensions of our spiral devices, only particles bigger than 13 μL can be focused on a streamline. In a blood sample, this should be translated into the presence of a remaining fraction of platelets and RBCs in the collection part corresponding to tumor cells. Despite this issue, the round spiral device allowed us to isolate and collect a high enriched fraction of big particles from the mid‐size ones when high density solutions (BSA at 30% and 16%) and asymmetrical flow rate were used.

### 2.4 Influence of flow solution density and flow rate on the isolation of osteosarcoma cells in round and spiral devices

It is generally assumed that tumor cells present higher size than blood cells, including WBCs.^42^, ^43^ When detached, standard osteosarcoma cell line models average in a round cell size of 17 μm of diameter (Table 1). The green fluorescent protein‐overexpressing MNNG‐HOS cell line (MNNG‐HOS) was used as a cell cancer model to analyze and quantify the cell distribution in our microfluidic spirals.

**TABLE 1:** Mean and standard deviation for surface and equivalent diameter for each OS cell line.

OS cell line	Surface (μm^2^)		EqDiameter (μm)	
	Media	SD	Media	SD
MNNG/HOS	243.78	74.94	17.42	2.6
KHOS	297.95	96.59	19.26	2.9
MG63	251.64	69.4	17.74	2.4
143B	189.96	35.76	15.48	1.44
SaOS‐2	228.74	69.04	16.9	2.35
U‐2 OS	202.5	45.66	15.96	1.73

**FIGURE 5:**
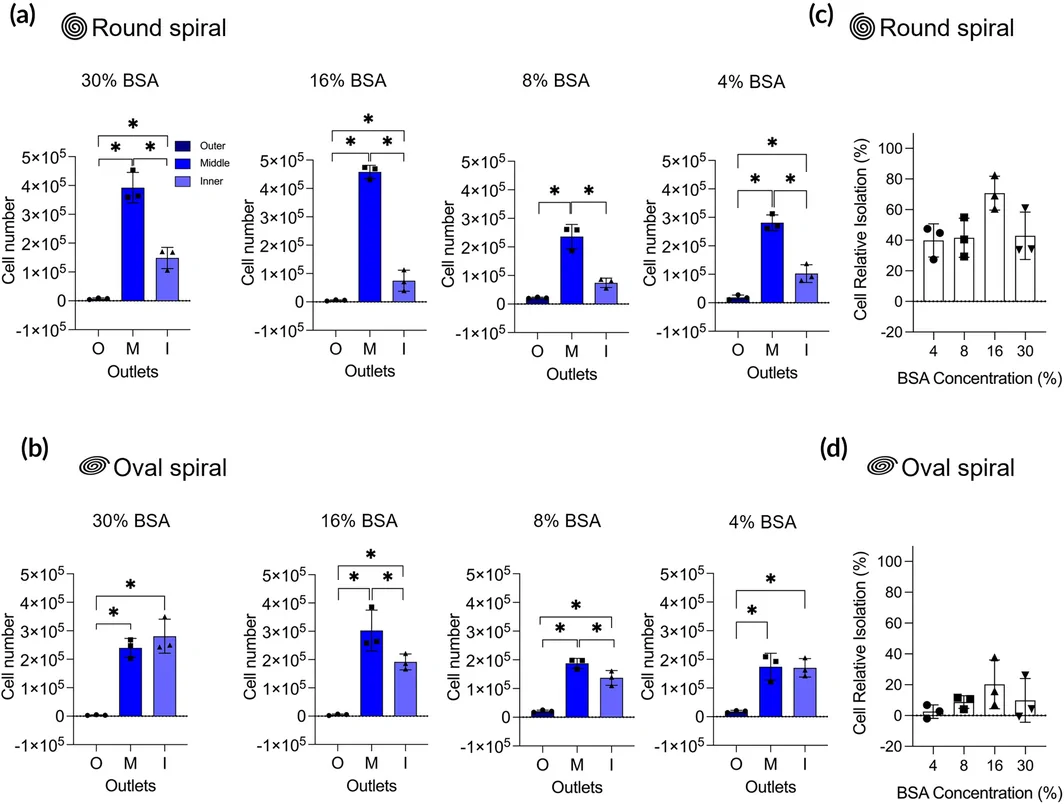
MNNG‐HOS cell distribution at different BSA concentrations in round and oval spirals at asymmetric flow rates. (a) Distribution of 500,000 GFP‐overexpressing MNNG‐HOS cells present in a solution of BSA at different concentrations into the round spiral microdevice and asymmetric flow rate. Cells collected at the different outlets were counted by using a fluorescent microscope and a specific macro for particle counting. Regardless of the density of the BSA solution, the MNNG‐HOS cells were significantly located at the medial outlet. (b) Distribution of 500,000 MNNG‐HOS cells in different concentrations of BSA subjected to asymmetrical flow rate in the oval spiral microdevice. Analyzed as (a), the presence of tumor cells was mainly focused in the middle and inner outlet regardless of the BSA concentration. (c) Quantification of relative isolation of MNNG‐HOS cells in the middle outlet from the round device at different BSA concentrations. For each test, data are expressed as the mean ± standard deviation of three independent experiments, analyzed using ordinary one‐way ANOVA and post hoc Holm‐Šídák's multiple comparisons test. *p‐value <0.05. O: outer outlet. M: middle outlet. I: inner outlet.

Like done for particle studies, individual solutions of BSA at different concentrations containing 500,000 MNNG‐HOS cells were used to challenge spiral microdevices with asymmetric flow rates. As shown in Figure 5a, osteosarcoma cells were recovered mainly from the middle outlet of the round spiral microdevices, regardless of the BSA concentration used. These results indicated that osteosarcoma cells presented an analogous distribution to the 13 μm particles and not as 26 μm particles, suggesting that in the round spiral to make cells got to the inner outlet cells should be at least bigger than 17 μm. Without excluding this hypothesis, another explanation could be the deformability of cells compared to solid particles when submitted to hydraulic forces influencing cell distribution^44^, ^45^ or that the intrinsic tumor cell size heterogeneity makes the full tumor cell isolation complicated (Table 1 and Ref. [46]). When cell solution was applied into the oval spiral, a balanced distribution of cells between the middle and the inner outlets was observed in all BSA concentrations (Figure 5b). This data agreed with the data obtained 13 μm particles (Figure 3b), suggesting that the presence of an enhanced second Dean drag force in the oval spiral favors the shift of the mid particles into the inner outlet.

**FIGURE 6:**
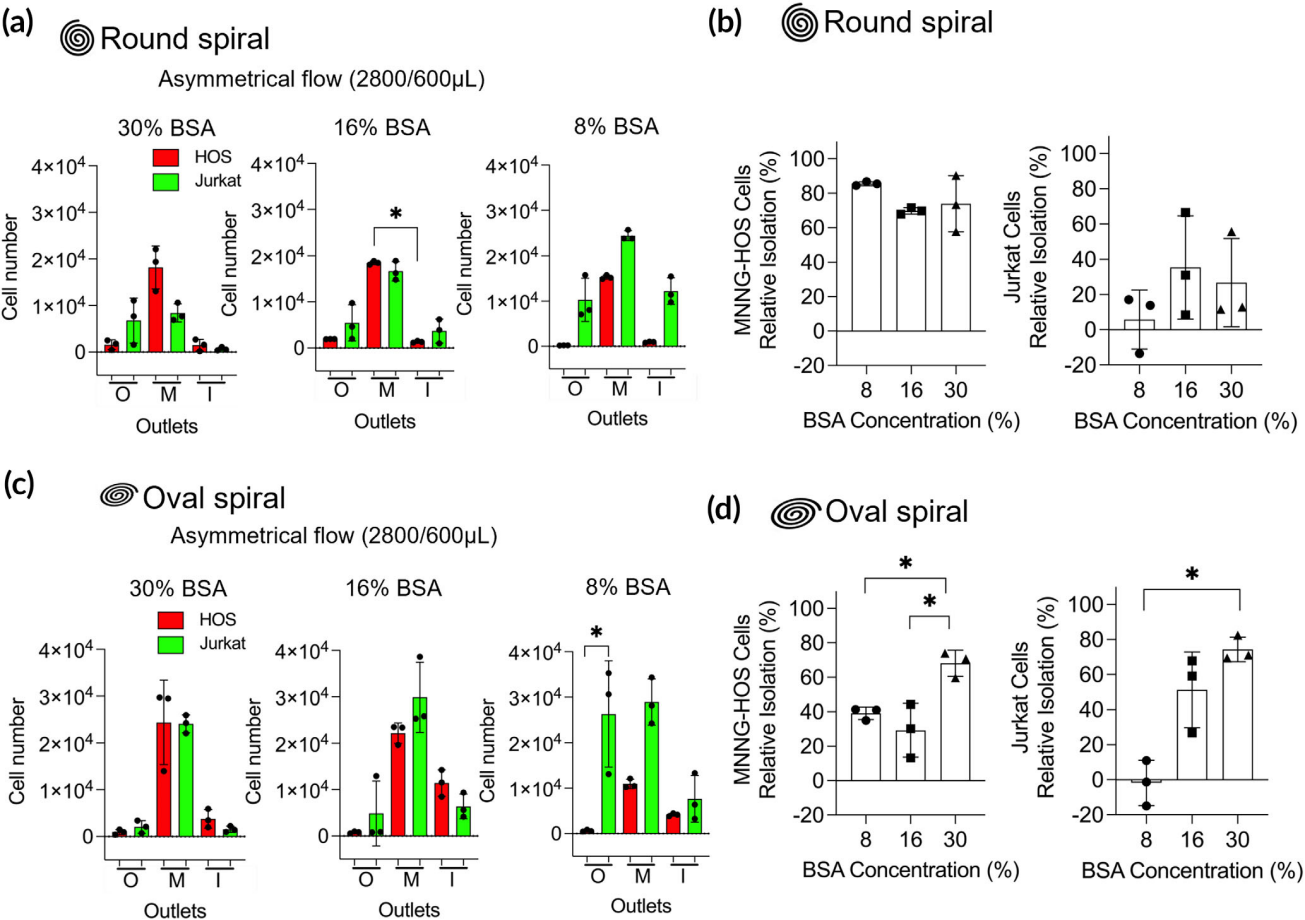
Mix of MNNG‐HOS and Jurkat cell distribution at different BSA concentrations in round and oval spirals at asymmetric flow rates. (a) Distribution of a mixture of 40,000 cells of each, MNNG‐HOS and Jurkat cells, at different BSA concentrations into the round spiral microdevice at asymmetric flow rate. Cells were collected at the different outlets and quantified by using a fluorescent microscope. Regardless of the density of the BSA solutions, the MNNG‐HOS cells (red bars) were mainly located at the middle outlet whereas Jurkat cells were mainly distributed between the outer and middle outlets (BSA 30 and 16%) or the three (BSA 8%). (b) Quantification of relative isolation of MNNG‐HOS and Jurkat cells in the main outlet (middle) of the round device at different BSA concentrations. (c) Distribution of a mixture of 40,000 cells of each, MNNG‐HOS and Jurkat cells, at different BSA concentrations into the oval spiral microdevice at asymmetric flow rate. Cell distribution was analyzed as (a). MNNG‐HOS cells (red bars) were mainly located in the middle outlet, while the Jurkat cells distribution (green bars) fluctuated between the outlets while decreasing the BSA density. (d) Quantification of relative isolation of MNNG‐HOS and Jurkat cells in the main outlet (middle) from the oval device at different BSA concentrations. For each test, data are expressed as the mean ± standard deviation of three independent experiments. The mixed‐size particles data were treated as paired and analyzed using the Friedman test and post hoc Dunn's multiple comparisons test; meanwhile, the relative isolation index was analyzed with ordinary one‐way ANOVA and post hoc Holm‐Šídák's multiple comparisons test or Kruskal–Wallis test with their corresponding post hoc Dunn's multicomparisons test when the parametric assumptions were not met. *p‐value <0.05. O: outer outlet. M: middle outlet. I: inner outlet.

To corroborate the data obtained by the particle mix solution, osteosarcoma cells were mixed with another population of cells which represented WBCs population with a different cell size than osteosarcoma cells, the Jurkat cell line, a human immortalized line of T lymphocyte cells. Those cells present an average size of 12 μm. To visualize, Jurkat cells were stained with HOECHST prior to cell mix preparation. Mix of 40,000 of each cell type were prepared with different BSA concentration (30%, 16% and 8%) and passed through the spiral devices (Figure 6). As shown in Figure 6a, the presence of Jurkat cells (green bars) did not alter the osteosarcoma cells distribution when applied into the round spiral, which was mainly recovered in the middle outlet (red bars). However, compared to results obtained for single MNNG‐HOS cell injection, the presence of Jurkat cells favored the focusing of osteosarcoma cells as shown by general increase of the relative isolation percentages (Figure 6b). Compared to single MNNG‐HOS data, the relative isolation index in BSA 30% was 73.85% ± 16.34% (vs. 42.89% ± 15.54%), 69.73% ± 1.91% (vs. 70.74% ± 11.05%) for BSA 16%, and 85.56% ± 1.14% (vs. 41.59% ± 12.81%) for BSA 8%. Interestingly, Jurkat cells presented similar behavior to particles of 10 μm in the mix solution (Figure 4), showing a distribution at the three outlets, with a main presence at the middle outlet (Figure 6a, green bars). Equivalent results were obtained when the mix of cells was passed through the oval spiral (Figure 6c). The distribution of MNNG‐HOS cells was mainly at the middle outlet (red bars) regardless of the percentage of BSA used. Thus, a reduction of the relative isolation index was observed for MNNG‐HOS cells (Figure 6d, left panel). In the case of Jurkat cells, the oval spiral allowed a better focusing of those cells into the middle outlet when a high percentage of BSA was used (Figure 6c, BSA 30% and 16%, green bars). Resulting in an increase in the relative isolation index in BSA 30% (74.31% ± 7.08%) compared to the obtained with the round spiral (26.74% ± 25.29%), as well as for BSA 16% (51.26% ± 21.62%) compared to the obtained with the round spiral (35.36% ± 29.27%) (Figure 6d, right panel).

**FIGURE 7:**
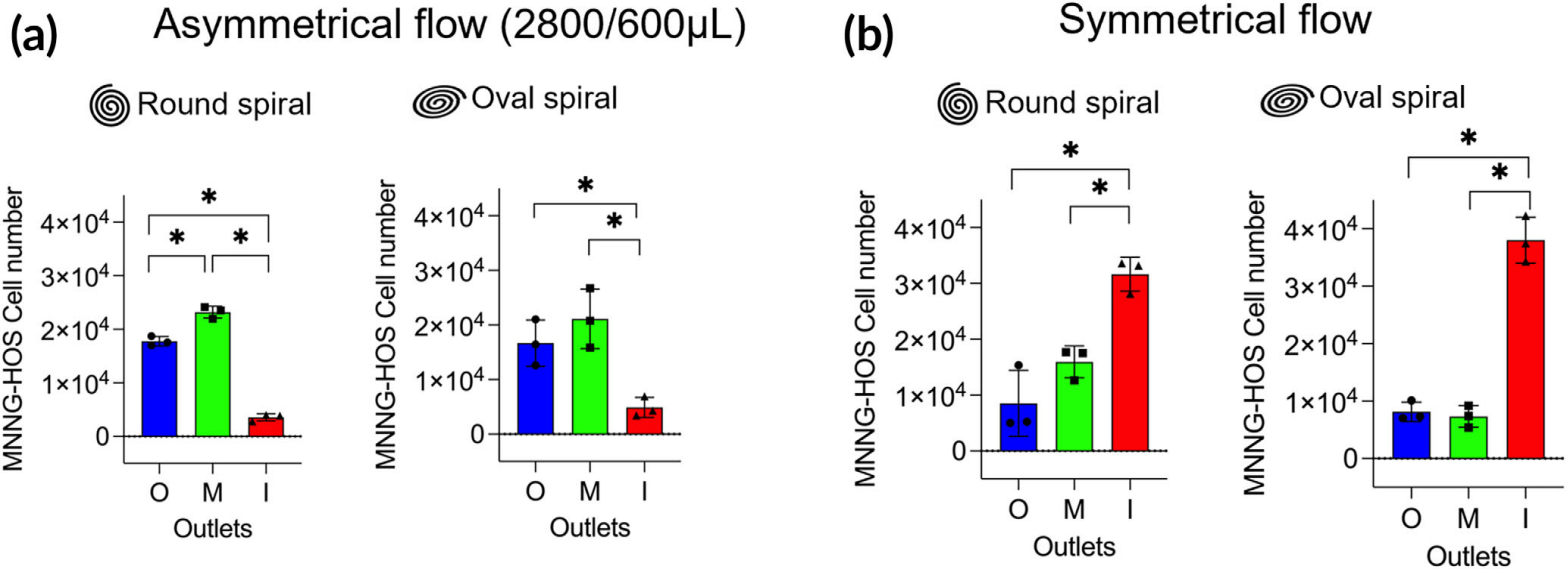
Distribution of MNNG‐HOS cells in blood at different flow rates and injection schemes in the round and oval spiral device. (a) Distribution of 40,000 MNNG‐HOS cells added into a 2.5 mL blood sample and injected into the round or oval spiral at asymmetric flow rate. GFP‐overexpressing MNNG‐HOS cells were collected and quantified at each outlet by fluorescent microscopy. Regardless of the spiral device, the MNNG‐HOS is surprisingly located within the middle and outer outlet, with a low presence in the inner outlet. (b) Distribution of 40,000 MNNG‐HOS cells added into a 2.5 mL blood sample and injected in the round or oval spiral at symmetric flow rate. MNNG‐HOS cells distribution was analyzed as (a). For each test, data are expressed as the mean ± standard deviation of three independent experiments. Data parametric assumptions were met and analyzed using ordinary one‐way ANOVA, followed by post hoc Holm‐Šídák's multiple comparisons test. *p‐value <0.05. O: outer outlet. M: middle outlet. I: inner outlet.

Previous data suggested that the increase of cells of different sizes and numbers could favor the focusing of tumor cells. We decided, then, to analyze the distribution of tumor cells when artificially added in a healthy blood sample. Blood samples containing MNNG‐HOS osteosarcoma cells (40,000 cells per 2.5 mL of blood) were injected through the round spiral with asymmetric flow rate. Unexpectedly, tumor cells were distributed between the middle (green bar) and outer (blue bar) outlets with a significant preference for the middle channel (23,207 ± 1106 cells and 17,775 ± 888.2 cells, respectively) (Figure 7a, left panel). A low number of cells was recovered in the inner outlet (red bar, 3577 ± 658 cells). This distribution of the osteosarcoma cell was the opposite of that observed in single osteosarcoma cell essays (Figure 5). Similar results were obtained in the oval spiral (Figure 7a, right panel). Significant differences were observed in cell recovery from the middle and outer outlets compared to the outer outlet (21,121 ± 658 cells and 16,671 ± 4217 cells vs. 4900 ± 1832). This inversion of distribution could be due to the extremely high number of cells in the blood sample and the use of asymmetric flow rate that could avoid the establishment of equilibrium forces in the spiral devices as already observed by Refs. [47, 48]. A reduction of the dominant flow rate could help to compensate dominant Dean drag forces and facilitate establishment of particles equilibrium in a determined position. We analyzed if initial symmetric flow rate tested (800 μL/min PBS and blood simultaneously) may help osteosarcoma cells refocusing in a blood solution. As shown in Figure 7b, a symmetrical flow rate relocated osteosarcoma cells significantly into the inner outlet when passed by the round spiral (Figure 7b, left panel). Under this condition, a significant fraction of cells was recovered into the inner outlet (31,657 ± 3032 cells) compared to the middle and outer outlets (15,954 ± 2857 and 8502 ± 5902 cells, respectively). Interestingly, the oval spiral resulted in a better distribution of osteosarcoma cells into the inner outlet (38,026 ± 4001 cells) than the round spiral (Figure 7b, right panel). Showing less distribution of cells into the middle and outer outlets (7320 ± 1900; 8141 ± 1691, respectively). Taking together, those data suggested that in the presence of high number of cell oval spiral microsystems with equally flow rates resulted in the best condition to isolate osteosarcoma cells from a blood sample; however, a considerable fraction of cancer cells were distributed also within middle and outer outlets.

**FIGURE 8:**
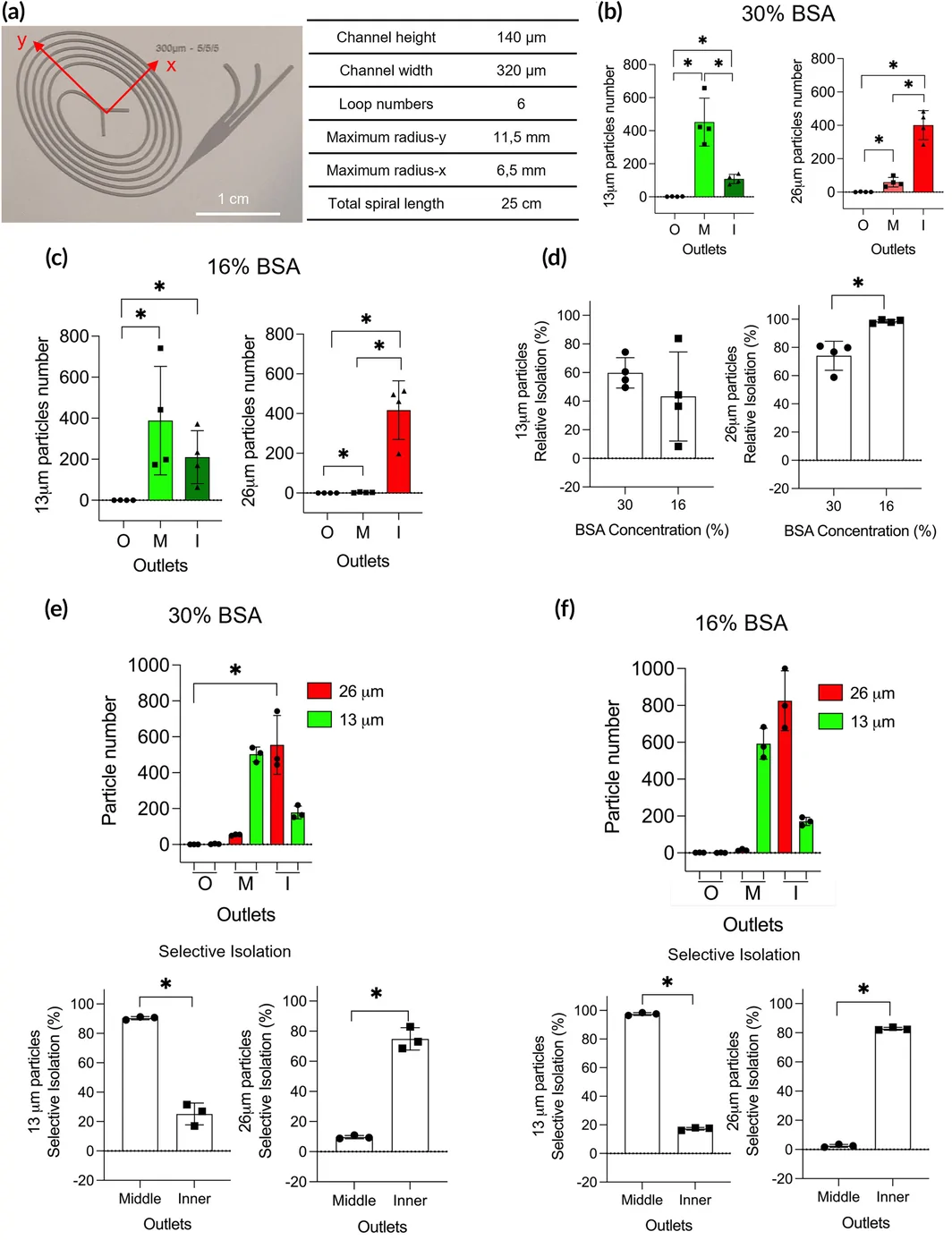
New oval microfluidic device. (a) Narrower oval spiral microsystem with six loop spirals in a counterclockwise direction and identical inlets and outlets exits than previous spirals. The main channel has a total length of 25 cm with a rectangular cross‐section measuring 320 μm in width and 140 μm in height resulting in a low aspect ratio of (*H*/*W* = 0.43). The short radius in the *x*‐axis has a size of 6.5 mm and the long radius in the *y*‐axis has a size of 11.5 mm. The loop spacing at the short *x*‐axis of the oval is 220 μm, whereas the long axis is 500 μm. Scale bar, 1 cm. (b) Distribution of 800 particles of 13 μm (green) or 26 μm (red) in BSA 30% through the new oval spiral at symmetric flow rate. For each individual solution, particles were collected from different outlets, analyzed and counted with a fluorescent microscope. (c) Distribution of 800 particles of 13 μm (green) or 26 μm (red) in BSA 16% through the new oval spiral at symmetric flow rate. Particles were analyzed as (b). (d) Quantification of relative isolation of 13 or 26 μm particles in BSA 30% and 16% through the narrower oval device. (e) Distribution and relative isolation of mixed‐size particles (800 particles of each size, 13 and 26 μm) in BSA 30% thought the new oval subjected to symmetrical flow rates. Particles were collected from different outlets, analyzed and counted with a fluorescent microscope. Top panel: 13 μm beads (green bars) were mainly collected at middle outlet whereas 26 μm beads (red bars) were mainly located into the inner outlet. Left bottom panel: comparative relative isolation of the 13 μm particles between the middle and the inner outlet. Right bottom panel: comparative relative isolation of the 26 μm particles between the middle and the inner outlet. (f) Distribution and relative isolation of mixed‐size particles (800 particles of each size, 13 and 26 μm) in BSA 16% thought the new oval subjected to symmetrical flow rates. Particles were collected from different outlets, analyzed and counted with a fluorescent microscope as in (e). Top panel: As similarly observed for BSA 30%, 13 μm beads (green bars) were mainly collected at middle outlet whereas 26 μm beads (red bars) were mainly located into the inner outlet. Left bottom panel: comparative relative isolation of the 13 μm particles between the middle and the inner outlet. Right bottom panel: comparative relative isolation of the 26 μm particles between the middle and the inner. For each single particle test, data are expressed as the mean ± standard deviation of four independent analyzed by unpaired *t*‐test or their nonparametric equivalent Mann–Whitney *U* test when the parametric assumptions were not met. For mixed‐size particles test, data are expressed as the mean ± standard deviation of three and treated as paired and analyzed using the Friedman test and post hoc Dunn's multiple comparisons test. The relative isolation index was analyzed with unpaired *t*‐test, and parametric assumptions were met. *p‐value <0.05. O: outer outlet. M: middle outlet. I: inner outlet.

Geometry and size of the spiral microchannel play a role in the distribution and focusing of particles of different sizes. Increasing the ratio between width and height can favor the focus of big particles out of a mixture of various sizes.^21^, ^49^ Based on the design of the oval spiral microsystem, a new version was generated by reducing the main channel width from 530 to 320 μm while preserving the channel height 140 μm (Figure 8a). This new system presented a height/width ratio of 0.43, which should increase the distance of focusing between the bigger particles.

We first analyzed the behavior of single particles (13 and 26 μm in BSA at 30%) through the new oval spiral device by symmetric flow rate. In comparison with the previous, wider oval spiral (Figure 2b, left panel), particles of 13 μm (left panel) were recovered mainly in the middle outlet, indicating that the new oval design induced a shift of 13 μm beads distribution from the inner outlet towards the middle outlet (Figure 8b, left panel). On the other hand, the position of 26 μm particles was unchanged with respect to the previous oval spiral (Figure 8b, right panel vs. Figure 2e, left panel). Reduction of BSA concentration to 16% did not improve the distribution of 13 μm particles into the middle outlet (Figure 8c, left panel). However, it favored the focusing of 26 μm particles into the inner outlet (Figure 8c, right panel). Regarding the recovery isolation index for those particles and BSA concentration in the new oval device, no significant differences were observed for 13 μm particles between BSA 30% and 16% (59.84% ± 10.62% and 43.27% ± 31.18%, respectively) (Figure 8d, left panel). But in the case of 26 μm particles, the solution at BSA 16% resulted in a significant increase in the relative isolation index compared to BSA 30% (98.48% ± 1.2% and 74.09% ± 10.31%, respectively) (Figure 8d, right panel).

When mixed solutions of 13 and 26 μm particles in BSA 30% (≈800 particles each) were injected into the new oval microsystem, each particle size behaved according to experiments with single particle solutions. Particles were sorted according to size with the 13 μm particles at the middle outlet and 26 μm particles exiting the inner outlet (Figure 8e, top panel). Comparing between the two outlets, the relative isolation index of 13 μm particles at the middle outlet (90.36% ± 1.2%) was significantly different than the one found in the inner outlet (25.14% ± 7.41%) (Figure 8e, left bottom panel). In the case of 26 μm particles, the relative isolation index at the inner outlet (74.86% ± 7.41%) was also significantly different than the one found in the middle outlet (9.64% ± 1.12%) (Figure 8e, right bottom panel). By decreasing the density of the mixed particle solution to BSA 16%, focusing was increased for each particle into the main collector outlet (Figure 8f, top panel). Interestingly, and unlike the single‐particle sorting, 13 μm particles did not increase their presence in the inner outlet. Moreover, a better relative isolation index at the main collector outlet was observed for each particle, being 97.48% ± 0.97% for 13 μm particles and 82.73% ± 0.96% for 26 μm particles (Figure 8f, bottom panels). This is also reflected in the fluorescent streamlines of the 13 μm (green) and 26 μm (red) particles (Figure S3). Compared with the previous wider oval design (Figure S3a,b, left panels), trajectories of 13 and 26 μm particles were better focused (Figure S3a,b, right panels). An important improvement was observed when using the BSA 16% solution. Clear, separated beams were obtained for both particles, with the 26 μm particle fluorescent streamline placed nearest the inner wall (Figure S3b, right panel).

**FIGURE 9:**
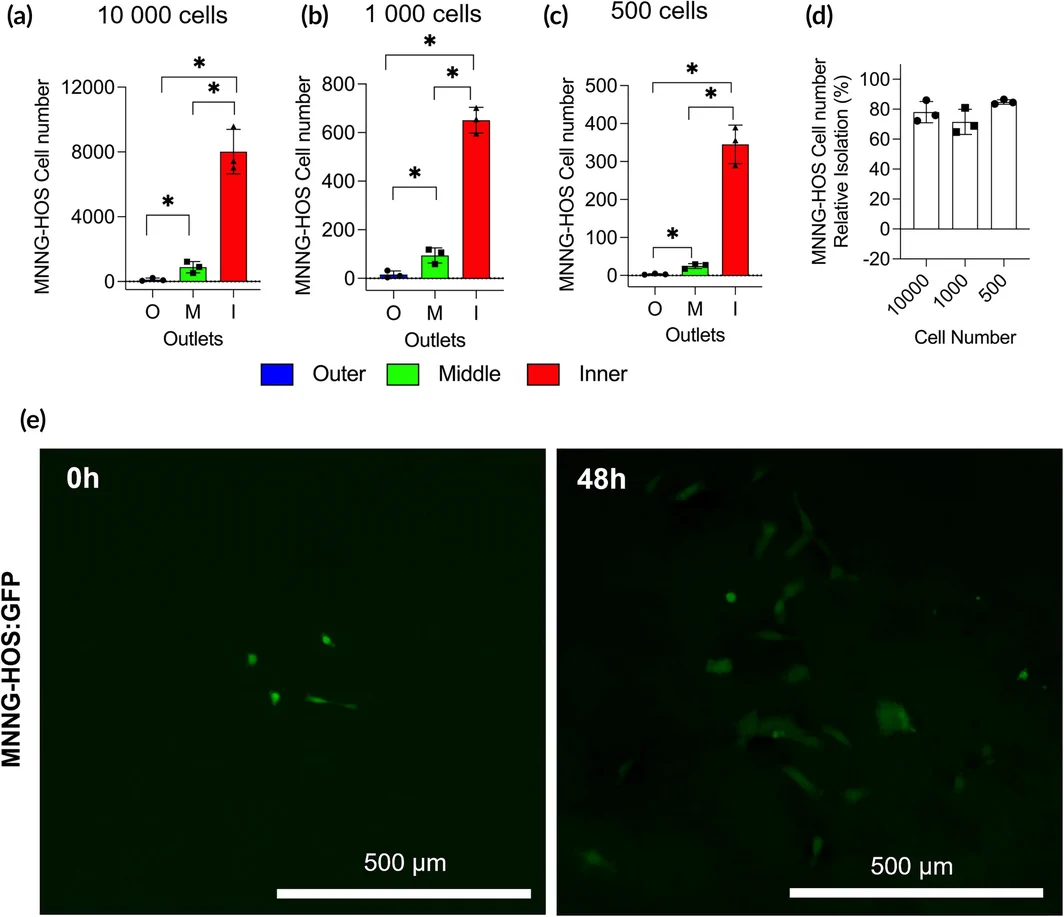
Distribution of different MNNG‐HOS cells quantities in blood sample in the narrower oval device at symmetric flow rate. (a) Distribution of 10,000 GFP‐overexpressing MNNG‐HOS cells added to a 2.5 mL blood sample. Cells were collected at the different outlets and counted by using a fluorescent microscope. (b) Distribution of 1000 MNNG‐HOS cells added to a 2.5 mL blood sample. Cells were analyzed as (a). (c) Distribution of 500 MNNG‐HOS cells added to a 2.5 mL blood sample. Cells were analyzed as (a). (d) Relative isolation of MNNG‐HOS from blood sample at the different cell quantities tested in (a)–(c). (e) MNNG‐HOS cells kept their proliferative properties after isolation. Tumor cells from the inner outlet were transferred into a 6 well plate and incubated 37°C incubator to analyze cell viability and proliferation Image are representative pictures of MNNG‐HOS cells at 0 and 48 h after isolation. For each test, data are expressed as the mean ± standard deviation of three independent experiments. The different MNNG‐HOS cells quantities met the parametric assumption and were analyzed using multiple *t*‐test. The relative isolation within the inner outlet met the parametric assumption and were analyzed using ordinary one‐way ANOVA, followed by post hoc Holm‐Šídák's multiple comparisons test. *p‐value <0.05. GFP channel image was obtained at 4x objective Nikon, Eclipse Ts2R‐FL, D‐LEDI. Scale bar: 500 μm. O: outer outlet. M: middle outlet. I: inner outlet.

Finally, we wondered if the fact that only a small fraction of middle size particles is distributed into the inner outlet of the new narrow (320 μm) oval spiral could be translated to the focusing of tumor cells into a single outlet (inner) (with a degree of) isolation from other blood cells. Then, blood samples containing 10,000 MNNG‐HOS osteosarcoma cells were tested in the new narrower oval spiral with symmetric flow rates. As shown in Figure 9a, osteosarcoma cells were significatively recovered in the inner outlet, resulting in a relative isolation index of 78. 07% ± 7.11% (Figure 9d). This result is consistent with that obtained in the 16% BSA solution (Figure 8c); this may be because this condition better represents the initial dilution of the whole blood sample with red blood cell lysis.

Moreover, a similar distribution to the inner outlet was observed when the number of osteosarcoma tumor cells was reduced in the blood samples to 1000 and 500 cells (Figure 9b,c). Interestingly, the relative isolation index was similar despite the reduction of osteosarcoma cell number: 71.52% ± 8.4% for 1000 cells and 84.83% ± 1.51% for 500 cells (Figure 9d). These data demonstrated the capacity of the new oval spiral in isolating a small number of tumor cells from blood samples. Moreover, in this allogenic blood spike model, fluorescent MNNG‐HOS cells were able to recover, culture, and keep proliferating after shorting (Figure 9e).

## 3 DISCUSSION

Early diagnosis of primary tumors or metastatic processes is a key step for the success of anti‐tumor therapies and good prognosis. Liquid biopsy is a minimally invasive and quick method widely used in medicine for detection of pathogens and altered molecules, metabolites, or cells. In this sense, the use and application of liquid biopsies in cancer is receiving particular attention nowadays.^6^ Within the different cancer elements that can be detected, CTCs have retained notable attention. Characterization of these cells constitutes valuable information about the tumor biology and therapy selection.^12^ However, detection and isolation of CTCs are a challenging process as they are sparsely present in blood. Several devices are commercially available for CTCs pre‐enrichment, such as Parsortix® or for sorting and isolation, Cell Search®, with a relatively high isolation and enrichment percentage.^50^, ^51^, ^52^ However, those technologies present several technical and economic limitations, including the numerous steps involved in sample processing and the overall cost of tests. To address these issues, microfluidic devices can constitute affordable, versatile, and accurate technology for cell sorting.^53^

Current microfluidics devices developed for particle or cell separation are based on active or passive methods.^54^ Active methods include acoustic, dielectrophoretic, magnetic, and optical sorting and imply the action of external forces that require specialized equipment and configurations that are associated with important cost. Whereas passive methods only rely on the action of fluid dynamics, including inertia, density, and various forces, simplifying their miniaturization and use. One such passive approach used for particle or cell separation is the use of an inertial focusing based spiral microsystem, where the interactions between inertial lift forces and Dean drag vortices determine the equilibrium position of particles in function of their size.^55^ Those hydrodynamic processes are strongly related to the spiral geometry and dimensions.^56^ The present work examines the influence of the spiral shape, flow rates, fluid density, and main channel cross‐section on particles and tumor cells distribution and isolation efficiency in spiral devices.

Several geometries have been used for cell sorting such as Archimedean, logarithmic, elliptical, conical, hyperbolic, and Fermat's shape. Using computational approaches, Shamloo and collaborators compared Archimedean, logarithmic, and elliptical shapes for theoretical isolation of CTCs. The three geometries showed good efficiency in CTCs isolation; however, the authors observed that elliptical spiral resulted in optimal and rapid organization of defined particle migration due to the formation of alternative Dean drag forces.^57^ Elliptical spiral devices are defined by two curvature radial axes (*x* and *y*). As shown by Erdem et al., the relation between the initial radius of both axes, known as initial aspect radius, is a parameter that favored the efficiency of particle separation.^58^ They analyzed the isolation efficiency of 20 and 10 μm particles using four elliptical spirals with different IAR (3:2, 11:9, 9:11, and 2:3). The four spirals resulted in particle purity close to 90%. The microsystem with the initial aspect radius of 3:2 was the one that produced the highest purity for both particles (95% and 90%). Our results align with these findings, showing that an elliptical shape generates better isolation efficiency than Archimedean, allowing a general isolation purity of ~90% for particles or cells above 13 μm.

Focusing of particles in straight channels is directly linked to the particle size and hydraulic diameter of the channel. As demonstrated by Di Carlo et al., for the focus of particles in a single beam the relation between the diameter of the particle and the channel hydraulic diameter (*a*_p_/*D*_h_) must be ≥0.07.^21^ For microchannel with a rectangular cross‐section, the hydraulic diameter is replaced by the channel height because of the shear rate variations across the channel.^59^ Our systems presented a channel height of 140 μm, what gives an *a*_p_/*D*_h_ closer to 0.07 for particles of 10 μm. This may explain the difficulties of our systems to focus the smaller particles and cells which are more influenced by the Dean drag forces. Moreover, the particle equilibrium is also affected by the aspect ratio of the cross‐section of the channel as shown by Nivedita et al.^60^ Our experimental data with particles and tumor cells showed that the increasing of the aspect ratio of the oval spiral from the initial design (*h*/*w* = 0.26) to the narrowed spiral (*h*/*w* = 0.43) resulted in a better focusing of particles. The fact of reducing channel width in rectangular cross‐section spiral may increase the action of lift forces favoring particles focusing as shown by Rodriguez‐Pena et al.^49^ These results aligned with recent publication by Oyilmaz and Gamze‐Ilis.^61^ which demonstrated by computational and experimental analysis that an increase of the cross‐section aspect‐ratio in rectangular spiral devices favored separation efficiency. In this sense, a promising approach is the use of a trapezoidal section spiral, where the aspect‐ratio is progressive, has been demonstrated that significantly improve the isolation particles as well as the recovery of CTCs as shown by several groups.^24^, ^30^, ^62^ However, design of this kind of geometry requires specific equipment and complex processing which is not widely accessible to researchers.

Flow rate also influences lateral particle migration and subsequently, particle separation as shown by Gao et al.^63^ General flow rate used in rectangular devices is around 2 mL/min, as summarized in Ref. [64]. Following global standard flow rates, in this work we have compared the effect of symmetrical (800 μL/min) and asymmetrical flow rates (600 and 2800 μL/min). Interestingly, whereas asymmetric flow rate resulted in the best condition for particle focusing and separation in discriminate outlets, when tumor cells were challenged, symmetric flow resulted in better focusing with a relative isolation index closer to 90%. This discrepancy between particle and cell behavior to the flow rate could be due to the deformability of cells compared to solid particles when submitted to hydraulic forces influencing cell distribution.^44^, ^45^ In addition, it may not be excluded that a reduction of the dominant flow rate could help to compensate dominant Dean drag forces as already described by Howell et al.^65^

Blood is a non‐Newtonian fluid that is generally modeled as a Newtonian fluid. This is an important aspect to consider when computational modeling or experimental essays are done in cell sorting from blood as, non‐Newtonian fluids display relevant differences hydrodynamic behaviors compared to Newtonian fluids.^66^ As demonstrated by Ref. [,^63^], viscosity plays an important role in lateral migration of particles. Several groups have developed sorting devices for particles and cell separation based in viscoelastic microfluidics.^67^ One of the main advantages of this approach is the use of untreated whole blood samples. The most used non‐Newtonian fluids are the poly(ethylene oxide) or PEO.^68^ Co‐flow injection of viscoelastic PEO solution with Newtonian samples resulted in the successful isolation of a low number of tumor cells from blood samples in straight channels.^69^ Using a similar microfluidics configuration, Shi et al. were able to isolate tumor cells from pleural effusion in a viscoelastic microfluidics characterized by the presence of a contraction−expansion array microchannel which provided the generation of Dean drag forces favoring parallel arrangement of big cells.^70^ As well as PEO, hyaluronic acid (HA) solution has been also used in viscoelastic microfluidics.^71^ By using a solution of 0.2% HA, Lim et al. were able to separate almost 95% of breast cancer cells from a mix of WBCs and a purity of 98%. In this work we have evaluated the impact fluid viscosity in particles and cells distribution in a non‐Newtonian solution based in BSA, the major protein present in blood.^72^ Our data showed that reduction of fluid viscosity to Newtonian parameters resulted in the misplacing of particles or cells focusing and, in particular, a wide distribution of smaller particles or cells. In the opposite way, isolation of tumor cells into a single outlet (inner) was favored at high concentration of BSA, reproducing the same behavior observed when cells were direct isolated from blood samples, and suggesting that BSA solutions can be used to mimic whole blood viscosity. This data is opposition with the observation made by Zhao et al^73^ They observed a dispersion of particles when resuspended in BSA solution following similar behavior than Newtonian fluids (water). One explanation to the particle dispersion may be the low concentration of BSA used as we observed similar particle dispersion when lower concentration of BSA were used (8% and 4%). Another explanation could be the different chip architecture. Zhao et al. experiments were done using straight rectangular microchannel and not spirals. It is possible that the Dean drags forces of the spiral in presence of high viscosity favor the alignment of big particles as observed by Shi et al. by the introduction in a straight microchannel the special module for generation of Dean Drags forces in viscoelastic microfluidics.^70^ Taking together these considerations, the use of non‐Newtonian fluids may be interesting methods to evaluate prototype microdevices based inertial focusing to better understand particle and cell behaviors.

Regarding blood in spiral microdevices, it is generally reported the pre‐treatment (erythrocyte lysis), pre‐enrichment (centrifugation) or dilution of blood samples prior injection into microdevices.^24^, ^31^, ^49^, ^60^, ^74^, ^75^, ^76^, ^77^ Whereas those treatments significantly improved separation efficiency of tumor cells by reducing the interaction of blood cells, they also may risk the loss of tumor cells from unique blood patient samples. Moreover, the level of dilution of blood samples is normally high from ten times to hundreds of times, which increases the volume of samples to pass by the microdevice and prolongs the time of processing (hours).^49^, ^74^, ^78^, ^79^ Here we have developed a microsystem with minimal direct manipulation of blood sample (see Section 4) which allowed whole blood sample loading and high recovery of tumor cells even when tested a low number. However, we also observed the presence of RBC in the purified fraction of isolated tumor cells. This issue is a common problem reported. Several groups have coupled several spirals to favor the cleaning of samples.^24^, ^75^ Another alternative is the use of microfluidics filters downstream.^80^, ^81^

This study has limitations that should be acknowledged. First, neither the internal pressure nor channel deformation was directly measured. Therefore, their potential influence on PDMS deformation and flow behavior could not be assessed. Second, polystyrene particles were used as a simplified model to evaluate the device performance under controlled conditions. Although it is useful for proof‐of‐concept validation, these particles do not fully reproduce the biological behavior of cells, their stiffness, deformability, buoyancy, and interaction with channel walls. Future studies should evaluate the device using more physiological models and stiffer materials, such as polymethyl methacrylate, to further validate the performance and robustness of the proposed design.

Despite the efficiency of inertial microfluidic platforms, one of the main issues in the field is the total purification of CTCs from blood or other fluids. Tumor cell heterogeneity implies the presence of cells of different sizes within the same sample.^46^ Additionally, depending on the tumor type, the size of CTCs also change.^82^ Generation of a universal CTCs isolation system that excludes potential contamination by blood cells remains a challenge. Furthers research will be needed for optimization of sensitivity and specificity in inertial microfluidic platforms. Implementation with other omics approaches may also be explored. Finally, the use of liquid biopsy remains a promising tool for deciphering tumor properties and biomarkers that may help for early cancer detection and development of personalized medicine therapies.

## 4 MATERIALS AND METHODS

### 4.1 Microfluidic devices: design and fabrication

Based on a spiral microchannel architecture, two microfluidic devices have been designed with an oval and round shape (Figure 1). Both devices consist of six loops spiral in the counterclockwise direction with two inlets at the center of the microchannel and three outlets outside in a perpendicular position with respect to the inlets. The two designs have a rectangular cross‐section channel, with a total width of 530 and 140 μm height giving a low aspect ratio (*H*/*W* = 0.26). The spiral of the round shape device presents a loop spacing of 250 μm, with a full length of 21 cm and an external radius of 6 mm. The oval spiral has a full length of 25 cm with a short radius in the *x*‐axis of 6.5 mm and a long radius in the *y*‐axis of 11.5 mm. The loop spacing at the short axis is 250 and 525 μm at the long axis. The new version of the oval design consists of a reduction of the main channel width to 320 μm instead of 530 μm, obtaining an aspect ratio higher than previous spirals (*H*/*W* = 0.43), but keeping the same height (140 μm) and exits architecture. The two inlets localized at the center part of the spiral have a length of 2 mm each, with the same width and height as the main channel. The exit of the spiral has a rectangular conical shape that finishes in three outlets channels. Its rectangular cross‐section has 140 μm of height with a starting width of 500 μm at the level of the spiral channel that reaches 2 mm at the outlets part. The outlets have similar cross‐section and size compared to the main spiral channel. The full length of the exits is 1.6 cm, including outlets.

The master mold was fabricated using SU‐82075 epoxy photoresist (Kayaku AM) by standard soft‐photolithography protocol.^83^ The spiral microchannels were fabricated in PDMS polymer (SYLGARD™ 184 Silicone Elastomer Kit, Dow Corning) in a 10:1 ratio with the curing agent. Mix solution was degassed, poured into the corresponding master mold, and baked at 65°C for at least 2 h in an oven (B121.0638, MEMMERT). Then PDMS microsystem was peeled off, and fluid access holes were made by biopsy punches of 1.5 mm (48115, BPP‐15F, Kai Medical). Finally, PDMS was bound to a microscope slide (VBS653/A, Biosigma) using an air plasma unit (PDC‐32G‐2/PDC‐VCG‐2, Harrick Plasma), and to ensure full sealing of the system, it was post‐baked for another 2 h at 65°C.

### 4.2 Experimental setup and procedure

To evaluate the efficacy of spirals in particle separation, three fluorescent polystyrene microspheres suspensions with different sizes, 10 μm (blue, F8829, Fluoro‐Max, Thermo Fischer Scientific®), 13 μm (green, 35‐4B, Fluoro‐Max, Thermo Fischer Scientific®), and 26 μm (red, 36‐5B, Fluoro‐Max, Thermo Fischer Scientific®) beads were used as references for blood cell populations. Ten micrometers particles represented the upper limit of lower cell size present in blood as platelets and RBC, 13 μm particles represented average cell size of WBC, and 26 μm particles represented higher cell size fraction (including CTCs). Final suspension for each particle was standardized in each volume for each experiment. The prepared beads suspension was fabricated using bovine serum albumin (BSA) (A7030, Sigma‐Aldrich) powder dissolved in PBS 1× (Dulbecco®) and filtered (0.2 μm filter) (Whatman Puradisc™ 25 mm PES Syringe Filters 6780‐2502). We used different concentrations of BSA to mimic the density and viscosity of the blood (1060 kg/m^3^ and 0.0035 Pa⋅s, respectively). 30% of BSA represented unaltered blood sample with similar density and viscosity.^35^ To mimic the dilutions factor of the whole blood volume, BSA solutions of different concentrations (16%, 8% and 4%) were used. Particle solutions or blood sample were driven through the system by using a 10 mL syringe (307736, BD Emerald®), which was subsequently loaded onto a programmable syringe pump (SPC/ZU‐I Independent Syringe Pump, Schenchen Boading®) (Figure S1). To favor particles or cells distribution, a second 10 mL syringe with PBS was loaded simultaneously into the microchannel allowing the dilution of the BSA sample within the microsystem. Based on previous publications^30^, we selected two different flow rate injections, a simultaneous injection at 800 μL/min and an asymmetrical injection of 2800 μL/min for the syringe containing PBS and 600 μL/min for the beads solutions. To inject the solution, PTFE tubes (BL‐PTFE‐1605‐20, Darwin Microfluidics®) of 20 cm were connected into the inlets and Tygon tubes (SA‐AAD04103‐15, ND 100‐80 Tubing, Darwin Microfluidics®) of 13 cm were placed into the outlets. Samples from each outlet tube were collected and visualized by using the Operetta® CLS™ High‐Content Analysis System fluorescent microscope (Revvity, Waltham, MA, USA). Particles fluorescence and size were analyzed using Harmony™ software (Revvity, Waltham, MA, USA). Video tracking of fluorescent particles or labeled cells were performed using a Nikon, Eclipse Ts2R‐FL, D‐LEDI coupled with MOMENT MONO camera (A24G635005).

### 4.3 Cell culture

Several tumor cell lines were used to mimic CTC behavior of different cell sizes in blood. Human MNNG/HOS (RRID: CVCL_0439, ATCC, cat number CRL‐1547) and 143‐B (RRID: CVCL_2270, ATCC, cat number CRL‐8303) osteosarcoma cell lines were cultured in Dulbecco's Modified Eagle's Medium (DMEM, Lonza, Levallois‐Perret, France) supplemented with 10% fetal bovine serum (FBS; Hyclone Perbio, Bezons, France) and 2 mmol/L of L‐glutamine. KHOS (RRID: CVCL_2546, ATCC, cat number CRL‐1544) and MG‐63 (RRID: CVCL_0426, ATCC, cat number CRL‐1427) osteosarcoma cell lines were cultured in Eagle's Minimum Essential Medium (EMEM, ref. M4655, Sigma‐Aldrich) supplemented with 10% FBS and 2 mmol/L of L‐glutamine. Saos‐2 (RRID: CVCL_0548, ATCC, cat number HTB‐85) and U2OS (RRID: CVCL_0042, ATCC, cat number HTB‐96) osteosarcoma cell lines were cultured in McCoy's 5a Medium (Gibco™ McCoy's 5a modified, ref. 16600082, Thermo Fisher Scientific) supplemented with 10% FBS and 2 mmol/L of L‐glutamine. Jurkat, Clone E6‐1, cell line (RRID: CVCL_0065, ATCC, cat number TIB‐152) was cultured in RPMI‐1640 Medium (Gibco™ RPMI 1640 Medium, GlutaMAX™ Supplemented, ref. 61870010, Thermo Fisher Scientific) supplemented with 10% FBS. All cells were cultured in a 5% CO_2_, 37°C incubator and cell detachment was done using Versene solution (ref. 15040066, Gibco™, Thermo Fisher Scientific). All the experiments using cell lines were performed between passages 2 and 4. All cell lines were regularly tested for the absence of mycoplasma.

### 4.4 Blood samples preparation

Blood from healthy human donors from the French National Blood Service (EFS convention CPDL‐PLER‐2021 050) was used for study particles and tumor cell separation in both spiral microchannel devices.

To avoid cell and platelet adhesion, tubes and spiral microchannels were first treated with Pluronic® F‐127 at 2% (102771094, Sigma Aldrich) during 1 h at room temperature and then washed with PBS. Then, to avoid blood coagulation, 50 μL of 1.5% Heparin Sodium Salt from Porcine solution (≥ 180 USP units/mg, H4784‐1G, Sigma‐Aldrich) was added to the tube containing 7 mL of blood, vortexed, and incubated at room temperature for 5 min. This treatment was reinforced by the addition of 500 μL of Sodium Citrate 3.2% (0.109 M) (Sodium citrate tribasic dihydrate, S4641‐500G, Sigma‐Aldrich), which sequestrates extracellular calcium and therefore affects calcium signaling in platelets, affecting platelet aggregation. Sample was vortexed and incubated at room temperature for 5 min. Finally, to reduce the number of blood cells, 2 mL red blood cell lysis solution (10×) (130‐094‐183, Miltenyi Biotec) was added to the blood sample, gently mixed, and incubated at room temperature for 15 min. No cell death on trypan blue staining and no loss of fluorescence was observed before or after in osteosarcoma cells included in whole‐blood samples undergoing red blood cell lysis.

### 4.5 Statistical analysis

The data analysis and visualization were performed using GraphPad Prism 8.0.2 (263) (GraphPad Software, San Diego, CA, EE. UU). Selected tests are required in figure legends or in the application. The choice of the statistical test depended on the experimental design and data characteristics.

To analyze the effect of a single particle size, data normality was assessed. Depending on whether parametric assumptions were met, either a multiple *t*‐test or a non‐parametric Mann–Whitney *U* test for independent measures was applied. To evaluate the effect of the mixture of two particle sizes simultaneously within the same sample, the data was treated as paired and analyzed using Friedman test with post hoc Dunn's multiple comparisons. For the relative isolation efficiency, either unpaired *t*‐test or ordinary one‐way ANOVA with post hoc Holm‐Šídák's multiple comparisons test was applied. When parametric assumptions were not met, Mann–Whitney *U* test or Kruskal–Wallis test with Dunn's multiple comparisons test were applied, respectively. Results are expressed in mean ± standard deviation or median with interquartile range and a *p*‐value ≤0.05 was considered statistically significant.

## AUTHOR CONTRIBUTIONS

**Isidora Panez‐Toro:** Investigation; writing – original draft; formal analysis; methodology; visualization. **Laurent Griscom:** Conceptualization; investigation; writing – review and editing; methodology; data curation; software. **Dominique Heymann:** Writing – review and editing; data curation. **Javier Muñoz‐García:** Conceptualization; writing – original draft; validation; writing – review and editing; project administration; supervision; data curation; funding acquisition; resources.

## FUNDING INFORMATION

The present work was supported by the Fondation de l'Avenir AP‐MR‐23‐021 (Recipient: J. Muñoz‐Garcia). Isidora Panez‐Toro PhD fellowship was supported by Region des Pays de la Loire and Institut de Cancérologie de l'Ouest.

## CONFLICT OF INTEREST STATEMENT

The authors declare no conflicts of interest.

## Supporting information


**Figure S1.** Experimental setup. Blood sample (or solution containing particles) and PBS solution are loaded onto 10 mL syringes and placed in programmable syringe pump (SPC/ZU‐I Independent Syringe Pump, Schenchen Boading®). Controlled flow rates are injected into the spirals devices thought PTFE tubes (BL‐PTFE‐1605‐20, Darwin Microfluidics®) of 20 cm connected into the inlets. Resulting samples are collected from each corresponding outlet into 15 mL Falcon tubes or well‐plates (6 or 96 well plates) via Tygon tubes (SA‐AAD04103‐15, ND 100‐80 Tubing, Darwin Microfluidics®) of 13 cm.

**Figure S2.** Efficiency isolation index of each particle size from the mixture‐size particle in different BSA concentrations and spiral designs. (a) Relative isolation of 10, 13 and 26 μm particles in the round spiral in different BSA concentrations. Left panel: the 10 μm particles relative isolation index for BSA 4% was 9% ± 18.05%, for BSA 8% 0.38% ± 3.67%, for BSA 16% 30.21% ± 11.51% and, for BSA 30% 33.83% ± 16.04%. Mid panel: the relative isolation index for 13 μm particles in BSA 4% was 41.63% ± 29.07%, for BSA 8% 61.41% ± 27.39%, for BSA 16% 74.90% ± 7.79% and, for BSA 30% 82.77% ± 10.66%. Right panel: 26 μm particle relative isolation index for BSA 4% was 98.48% ± 2.56%, for BSA 8% 98.05% ± 1.50%, for BSA 16% 89.90% ± 12.47% and, for BSA 30% 74.70% ± 21.95%. (b) Relative isolation of 10, 13 and 26 μm particles in the oval spiral in different BSA concentrations. Left panel: the 10 μm particles relative isolation index for BSA 4% was 10% ± 6.76%, for BSA 8% 15.51% ± 18.39%, for BSA 16% 28.73% ± 13.31%, and, for BSA 30% 32.52% ± 26.66%. Mid panel: the relative isolation index for 13 μm particles in BSA 4% was 29.46% ± 19.40%, for BSA 8% 11.03% ± 10.36%, for BSA 16% 35.79% ± 25.6% and, for BSA 30% 35.12% ± 22.60%. Right panel: 26 μm particle relative isolation index for BSA 4% was 96.37% ± 2.44%, for BSA 8% 97.85% ± 1.54%, for BSA 16% 98.61% ± 0.78% and, for BSA 30% 96.14% ± 3.23%. For each test, data are expressed as the mean ± standard deviation of four independent experiments. All mixed‐size particle distributions were analyzed using ordinary one‐way ANOVA and post hoc Holm‐Šídák's multiple comparisons test. *p‐value <0.05.

**Figure S3.** Fluorescent streamline comparison distribution of 26 μm and 13 μm mixed particles in BSA 30% and 16% between the oval and narrower oval design. (a) Distribution of mixed 13 and 26 μm particles subjected to symmetrical flow rates in the oval and new narrower oval spiral microdevice. 2.5 mL of a solution containing 800 particles one particle size in BSA 30% was injected simultaneously with 2.5 mL of PBS into the round spiral at an equal flow rate of 800 μL/min. Meanwhile the system was running, images of the fluorescent streamline were obtained in merged GFP and CY3 channel to respective particle fluorescence. In comparison with the wider oval device, once is reduced the width the 13 μm particles fluorescent streamline is displacing towards the middle outlet being represented also within their respective graph above. (b) With the same set‐up in (a) except for the BSA 16%, images were obtained and with similar fluorescent streamline distribution it is observed that narrowing the oval device the red 26 μm particles streamline was nearest the inner wall with a green 13 μm particles streamline than their equivalent at BSA30%. GFP and CY3 merged images were obtained at 4× objective Nikon, Eclipse Ts2R‐FL, D‐LEDI. Scale bar: 500 μm.

## Data Availability

The main data generated and analyzed during this study are included in this published article and its supplementary information files. However, complementary datasets generated during and/or analyzed during the current study are also available from the corresponding author on reasonable request.
